# Supplementary material for: Effects of Probiotics on Autism Spectrum Disorder in Children: A Systematic Review and Meta-Analysis of Clinical Trials
Source: Nutrients. 2023 Mar 15;15(6):1415. doi: 10.3390/nu15061415 (PMC10054498; doi:10.3390/nu15061415)
Supplement: Supplementary file 1 [file nutrients-15-01415-s001.zip › nutrients-2246022-supplementary.pdf]

Supplementary File S1

Table S1 The detailed search strategies

Database	Search items	Simple size
CNKI	(“autism spectrum disorder” OR “ASD” OR “autism” OR “autistic disorder” OR “asperger syndrome” OR “asperger disorder” OR “autistic traits”) AND (“microbiota” OR “microbiome” OR “microflora” OR “probiotic” OR “probiotics”)	11
ScienceDirect	(“autism spectrum disorder”) AND (“probiotic”) AND (“children”)	170
PubMed	("autism spectrum disorder"[MeSH Terms]) AND ("probiotics"[MeSH Terms])	81
Web of Science	(“autism spectrum disorder”) AND (“children”)	224
Cochrane library	(“autism spectrum disorder”) AND (“probiotic”) AND (“children”)	45
Medalink	(“autism spectrum disorder”) AND (“probiotic”) AND (“children”)	145

ScienceDirect:

Use its built-in filtering function in ScienceDirect and only keep studies with a research type of “Research articles”: 170

Web of Science:

search in the results USING (“microbiota” OR “microbiome” OR “microflora” OR “probiotic” OR “probiotics”). Then, use its built-in filtering function in web of science to filter out studies with study types “Abstract”, “Meeting”, “News”, “Letter”, “Biography”, “Book”, “Correction”, “Unspecified”, “Editorial Material”, “Case Report”, “Early Acess”, “Other”, “Review Article”: 224

Supplementary File S2

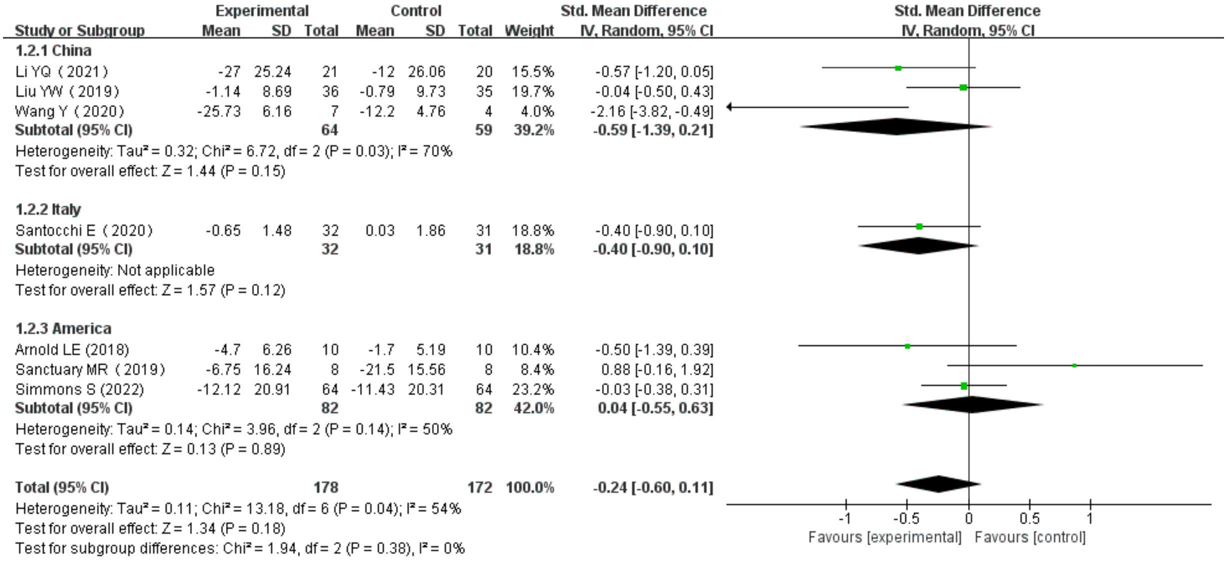

Figure S1 Forest plot for the subgroup analysis of country [1–7].

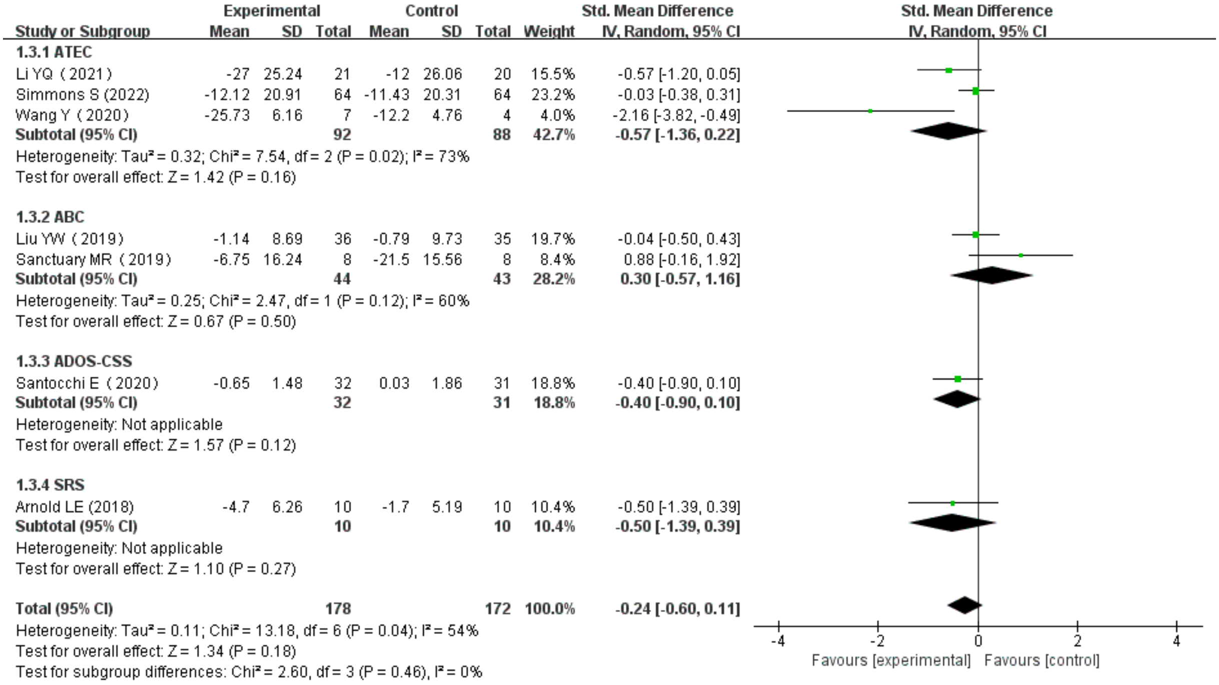

Figure S2 Forest plot for the subgroup analysis of scale [1–7].

Study or Subgroup	Experimental Mean	Experimental SD	Experimental Total	Control Mean	Control SD	Control Total	Weight	Std. Mean Difference IV, Random, 95% CI
**1.4.1 ＜3 months**								
Arnold LE (2018)	-4.7	6.26	10	-1.7	5.19	10	10.4%	-0.50 [-1.39, 0.39]
Li YQ（2021）	-27	25.24	21	-12	26.06	20	15.5%	-0.57 [-1.20, 0.05]
Liu YW（2019）	-1.14	8.69	36	-0.79	9.73	35	19.7%	-0.04 [-0.50, 0.43]
Sanctuary MR（2019）	-6.75	16.24	8	-21.5	15.56	8	8.4%	0.88 [-0.16, 1.92]
Simmons S (2022)	-12.12	20.91	64	-11.43	20.31	64	23.2%	-0.03 [-0.38, 0.31]
**Subtotal (95% CI)**			**139**			**137**	**77.2%**	**-0.11 [-0.45, 0.23]**
Heterogeneity: Tau² = 0.06; Chi² = 6.56, df = 4 (P = 0.16); I² = 39%								
Test for overall effect: Z = 0.63 (P = 0.53)								
**1.4.2 ＞3 months**								
Santocchi E（2020）	-0.65	1.48	32	0.03	1.86	31	18.8%	-0.40 [-0.90, 0.10]
Wang Y（2020）	-25.73	6.16	7	-12.2	4.76	4	4.0%	-2.16 [-3.82, -0.49]
**Subtotal (95% CI)**			**39**			**35**	**22.8%**	**-1.09 [-2.78, 0.59]**
Heterogeneity: Tau² = 1.15; Chi² = 3.92, df = 1 (P = 0.05); I² = 75%								
Test for overall effect: Z = 1.27 (P = 0.20)								
**Total (95% CI)**			**178**			**172**	**100.0%**	**-0.24 [-0.60, 0.11]**
Heterogeneity: Tau² = 0.11; Chi² = 13.18, df = 6 (P = 0.04); I² = 54%								
Test for overall effect: Z = 1.34 (P = 0.18)								
Test for subgroup differences: Chi² = 1.26, df = 1 (P = 0.26), I² = 20.6%								

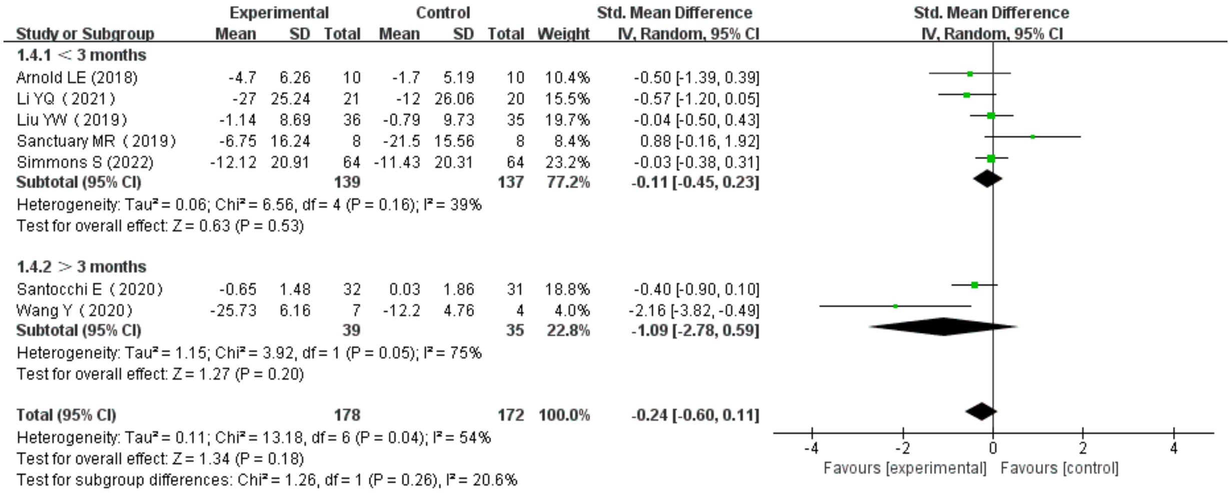

Figure S3 Forest plot for the subgroup analysis of duration of intervention [1–7].

Supplementary File S3

```
. metabias6 _ES _seES

Note: default data input format (theta, se_theta) assumed.

Tests for Publication Bias

Begg's Test

  adj. Kendall's Score (P-Q) =      -7
          Std. Dev. of Score =    6.66
           Number of Studies =       7
                          z  =   -1.05
                    Pr > |z| =   0.293
                          z  =    0.90 (continuity corrected)
                    Pr > |z| =   0.368 (continuity corrected)

Egger's test
```

Std_Eff	Coef.	Std. Err.	t	P>|t|	[95% Conf.	Interval]
slope	.155804	.3930514	0.40	0.708	-.8545668	1.166175
bias	-1.307544	1.367213	-0.96	0.383	-4.822077	2.206989

Figure S4 Tests for Publication Bias [1–7].

Supplementary File S4

Study omitted	Estimate	[95% Conf.	Interval]
Li YQ (2021)	-.18491553	-.58376527	.21393423
Liu YW (2019)	-.30536348	-.7535603	.14283332
Santocchi E (2020)	-.21827218	-.65236002	.21581562
Wang Y (2020)	-.16650178	-.45337558	.12037205
Arnold LE (2018)	-.21696721	-.61272627	.17879187
Sanctuary MR (2019)	-.31627268	-.639	.00645462
Simmons S (2022)	-.31604666	-.77837366	.14628035
Combined	-.24296432	-.599521	.11359237

Figure S5 Test of sensitivity analysis [1–7].

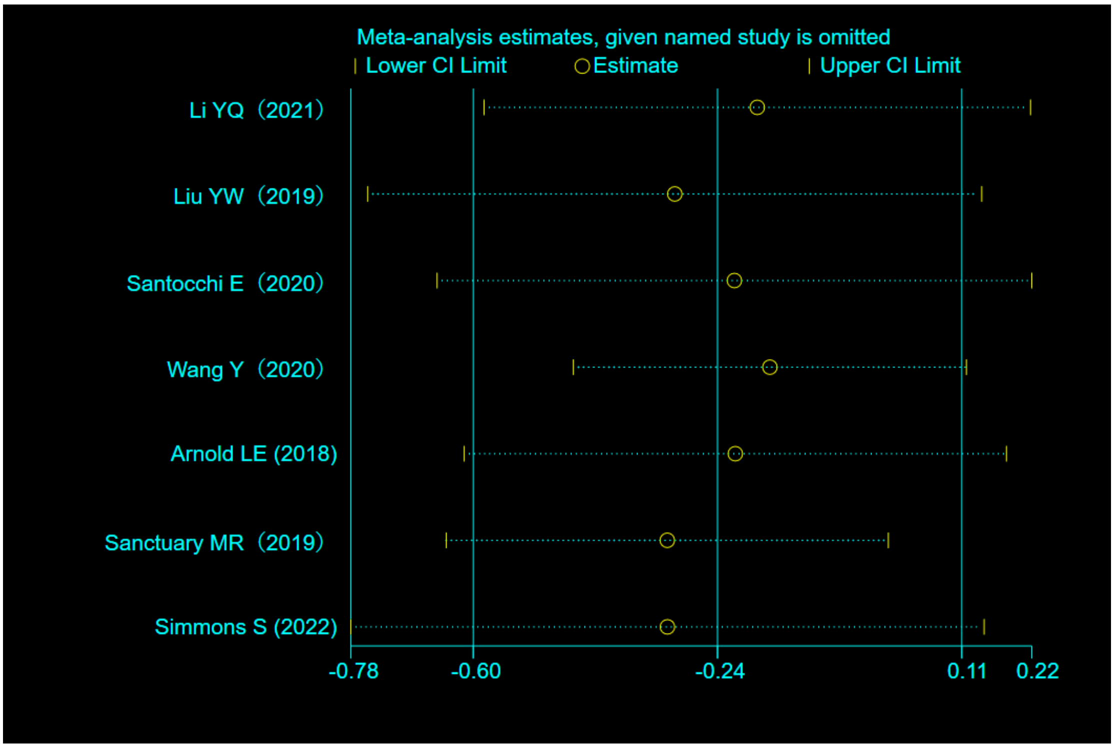

Figure S6 Plot of sensitivity analysis [1–7].

References

1. Liu, Y.W.; Liong, M.T.; Chung, Y.E.; Huang, H.Y.; Peng, W.S.; Cheng, Y.F.; Lin, Y.S.; Wu, Y.Y.; Tsai, Y.C. Effects of Lactobacillus plantarum PS128 on Children with Autism Spectrum Disorder in Taiwan: A Randomized, Double-Blind, Placebo-Controlled Trial. *Nutrients* **2019**, *11*, 820. https://doi.org/10.3390/nu11040820.
2. Li, Y.Q.; Sun, Y.H.; Liang, Y.P.; Zhou, F.; Yang, J.; Jin, S.L. Effect of probiotics combined with applied behavior analysis in the treatment of children with autism spectrum disorder: A prospective randomized controlled trial. *Zhongguo Dang Dai Er Ke Za Zhi* **2021**, *23*, 1103–1110. https://doi.org/10.7499/j.issn.1008-8830.2108085. (In Chinese)
3. Santocchi, E.; Guiducci, L.; Prosperi, M.; Calderoni, S.; Gaggini, M.; Apicella, F.; Tancredi, R.; Billeci, L.; Mastromarino, P.; Grossi, E.; et al. Effects of Probiotic Supplementation on Gastrointestinal, Sensory and Core Symptoms in Autism Spectrum Disorders: A Randomized Controlled Trial. *Front. Psychiatry* **2020**, *11*, 550593. https://doi.org/10.3389/fpsyt.2020.550593.
4. Arnold, L.E.; Luna, R.A.; Williams, K.; Chan, J.; Parker, R.A.; Wu, Q.; Hollway, J.A.; Jeffs, A.; Lu, F.; Coury, D.L.; et al. Probiotics for Gastrointestinal Symptoms and Quality of Life in Autism: A Placebo-Controlled Pilot Trial. *J. Child Adolesc. Psychopharmacol.* **2019**, *29*, 659–669. https://doi.org/10.1089/cap.2018.0156.

5. Sanctuary, M.R.; Kain, J.N.; Chen, S.Y.; Kalanetra, K.; Lemay, D.G.; Rose, D.R.; Yang, H.T.; Tancredi, D.J.; German, J.B.; Slupsky, C.M.; et al. Pilot study of probiotic/colostrum supplementation on gut function in children with autism and gastrointestinal symptoms. *PLoS ONE* **2019**, *14*, e0210064. https://doi.org/10.1371/journal.pone.0210064.
6. Available online: https://clinicaltrials.gov/ct2/show/results/NCT03369431 (accessed on 17 January 2023).
7. Wang, Y.; Li, N.; Yang, J.J.; Zhao, D.M.; Chen, B.; Zhang, G.Q.; Chen, S.; Cao, R.F.; Yu, H.; Zhao, C.Y.; et al. Probiotics and fructo-oligosaccharide intervention modulate the microbiota-gut brain axis to improve autism spectrum reducing also the hyper-serotonergic state and the dopamine metabolism disorder. *Pharmacol. Res.* **2020**, *157*, 104784. https://doi.org/10.1016/j.phrs.2020.104784.
